# Engineering a 3D matrix from porcine plasma for enhanced cell proliferation and differentiation

**DOI:** 10.3389/fvets.2026.1611532

**Published:** 2026-04-30

**Authors:** Yangyang Ma, Xiaohui Peng, Yuting Lin, Tiantian Xia, Qianqian Pan, Yuhang Wu, Mingjing Cui, Shuaifeng Shen, Fugui Fang, Yunsheng Li, Zubing Cao, Yunhai Zhang, Tong Yu

**Affiliations:** 1 College of Animal Science and Technology, Anhui Agricultural University, Hefei, China; 2 Anhui Province Key Laboratory of Local Livestock and Poultry Genetic Resource Conservation and Biobreeding, College of Animal Science and Technology, Anhui Agricultural University, Hefei, China; 3 Linquan Comprehensive Experimental Station of Anhui Agricultural University, Anhui Agricultural University, Linquan, China; 4 College of Veterinary Medicine, Anhui Agricultural University, Hefei, China

**Keywords:** 3D cell culture, cell proliferation, mesenchymal stem cells, platelet lysate, porcine fibrin matrix gel

## Abstract

**Introduction:**

The limited availability of suitable scaffolds for three-dimensional (3D) cell culture has driven the development of new biomaterials. In this study, we developed a porcine fibrin matrix gel (PFMG) derived entirely from porcine blood as a species-matched 3D scaffold that integrates the structural support of fibrin with the bioactive components of porcine platelet lysate (PPL).

**Methods:**

PFMG was prepared from porcine plasma supplemented with PPL and calcium to induce gelation. Its physicochemical properties, including tunable stiffness, fibrillar microstructure, and molecular sieve–like behavior, were characterized by coagulation testing, diffusion assays, scanning electron microscopy, and rheological analysis. The biological performance of PFMG was evaluated using porcine mesenchymal stem cells (MSCs) and porcine embryonic fibroblasts (PEFs), and compared with conventional two-dimensional (2D) cultures supplemented with fetal bovine serum (FBS) or PPL. Cell proliferation, MSC tri-lineage differentiation, and transcriptomic changes were assessed.

**Results:**

PFMG formed a stable fibrin-based 3D network with adjustable mechanical properties and selective permeability to small molecules. Compared with conventional 2D culture systems, PFMG significantly enhanced the proliferation of both MSCs and PEFs and supported a characteristic 3D cellular morphology. MSCs cultured in PFMG retained adipogenic, osteogenic, and chondrogenic differentiation potential, and adipogenic differentiation was further enhanced when induction was performed with PPL instead of FBS. Transcriptomic analysis of MSCs cultured in PFMG revealed a distinct gene-expression profile characterized by upregulation of genes involved in cell-cycle progression, oxidative phosphorylation, and growth-related pathways, alongside downregulation of genes associated with cell adhesion and extracellular matrix interactions.

**Discussion:**

These findings indicate that PFMG provides a supportive, species-specific, and cost-effective 3D microenvironment for porcine cell culture. By combining scaffold function with platelet-derived bioactivity, PFMG promotes cell expansion while preserving multilineage differentiation capacity. This fully porcine platform therefore represents a promising biomaterial for porcine cell culture, tissue engineering, and regenerative medicine applications.

## 1 Introduction

Tissue engineering and regenerative medicine have an urgent need for effective scaffolds capable of supporting cell growth and new tissue formation, especially given the critical shortage of donor tissues and organs. An ideal scaffold should emulate the native extracellular matrix, facilitating cell attachment, proliferation, and differentiation. Despite substantial progress in scaffold development, many synthetic materials suffer from poor biocompatibility, inadequate cell adhesion, or undesirable degradation rates. This has increased interest in natural polymeric scaffolds, particularly fibrin-based matrices, which offer excellent biocompatibility, controllable degradation kinetics, and robust support for cell–matrix interactions.

Fibrin is a natural biopolymer formed from blood plasma fibrinogen upon activation by thrombin, resulting in a 3D fibrous network that provides structural support for cell growth (1). Fibrin hydrogels have several advantages over synthetic scaffolds, including superior cytocompatibility and tunable biodegradation, and they have been widely used as cell carriers in tissue engineering applications. Notably, human plasma–derived fibrin scaffolds have been successfully applied in autologous skin grafts, skeletal muscle regeneration, and bone tissue repair, among other applications (2–5). Whereas human fibrin-based scaffolds are well studied, fibrin from animal sources (particularly porcine) offers a cost-effective and readily available alternative for biomaterial fabrication.

Providing appropriate biochemical cues in the cell culture environment is also crucial for tissue regeneration. Platelet-rich preparations are an abundant source of growth factors and cytokines involved in wound healing (6–8). Platelet lysate (PL), obtained from platelet-rich plasma (PRP), has emerged as an effective supplement for cell culture media, often outperforming fetal bovine serum (FBS) in promoting cell growth (9). In porcine cell culture systems, PPL similarly enhances cell proliferation (10), making it a promising alternative to FBS in veterinary and translational research. A key advantage of PPL lies in the vast and stable availability of porcine blood as a sustainable agricultural by-product. This high-yield resource allows for the cost-effective and scalable production of standardized supplements, overcoming the supply constraints often encountered with human-derived PL. Furthermore, as a species-matched (allogenic) supplement, PPL provides a more physiologically relevant biochemical environment for porcine cells compare to FBS. Consequently, PPL serves as an ideal foundation for building high-performance, species-specific culture platforms in the veterinary and regenerative research fields.

In this study, we address the need for a readily obtainable, growth factor–rich 3D scaffold by developing a porcine fibrin matrix gel (PFMG) derived entirely from pig blood. PFMG integrates the structural support of a fibrin network with the bioactive factors from platelets, effectively combining the benefits of a physical scaffold and a biochemical supplement in one platform. We characterized the physicochemical properties of PFMG (including its adjustable stiffness and molecular sieve–like behavior) and evaluated its ability to support the growth and multilineage differentiation of porcine MSCs and PEFs. The performance of PFMG-based cultures was compared to conventional 2D cultures supplemented with FBS or PPL. Our results demonstrate that PFMG provides a highly supportive 3D microenvironment for porcine cells, highlighting its potential as an innovative scaffold for tissue engineering, regenerative medicine, and cell therapy applications.

## 2 Materials and methods

### 2.1 Separation of porcine plasma and platelets

To prepare plasma and platelets, 123 mL of anticoagulant CPDA-1 was added in advance to a 1 L blood collection bottle and evenly distributed by gentle inversion to coat the interior surface. Fresh whole blood was collected from 12 healthy Duroc × Landrace × Yorkshire (DLY) pigs (~6 months old; equal numbers of males and females) into this bottle up to ~1 L and inverted five times to mix it with the anticoagulant. The anticoagulated blood was allowed to stand at 20–22 °C for 1 h, resulting in separation into three layers: a bottom layer of red blood cells (approximately 50–70% of the volume), a thin buffy coat containing platelets and white blood cells, and an upper layer of PRP. The top two-thirds of the PRP layer were carefully transferred into 15 mL centrifuge tubes and centrifuged at 3,200 rpm for 20 min at 21 °C. The supernatant (plasma) was collected for further use. The remaining platelet pellets were separated, transferred into cryogenic vials, weighed and labeled, then stored in liquid nitrogen for subsequent preparation of PPL. The plasma was sterilized through a 0.22 μm vacuum filter and stored at −80 °C for later use in producing the PFMG.

To assess the reproducibility of this blood component preparations, this entire separation and collection procedure was performed independently four times using consistent animal and handling conditions, and the resultant plasma and platelet fractions were processed in the same manner. Preliminary materials testing and cell-culture experiments confirmed that the physicochemical properties of the plasma, platelets and derived gels were highly reproducible across all batches and supported equivalent cell growth. Experiments and data presented in this manuscript are based on materials from one representative batch.

### 2.2 Preparation of PPL and PFMG

To minimize cytokine loss during plasma clotting, we induced coagulation of the prepared plasma and then removed the clot to produce porcine serum. This approach prevented excessive cytokine adsorption to fibrin and preserved the quality of the porcine serum. Because a citrate-based anticoagulant (CPDA-1) was used, we reintroduced calcium ions along with a small portion of the platelet concentrate to trigger coagulation. In this way, coagulation factors and Ca^2+^ were added to the plasma clotting process, allowing the plasma to clot without the adverse effects of excessive Ca^2+^ on cells. We determined the optimal Ca^2+^ amount through a titration experiment (see Table 1). Once the appropriate CaCl_2_ dosage was identified, CaCl_2_ was added to the plasma according to this titration (final Ca^2+^ concentration ~8 mmol/L), along with a small quantity of platelets (0.2 g/L), to coagulate a large volume of plasma. The plasma was left undisturbed at 37 °C for 40 min to form a solid clot (the prototype of PFMG). The plasma clot was then centrifuged at 9,000 × g for 30 min at 4 °C. The supernatant after centrifugation was collected as the fibrinogen-depleted porcine serum, which we termed the second-regenerated (2nd) serum.

**Table 1:** CaCl_2_ titration test.

Coagulation data	1#	2#	3#	4#	5#	6#	7#	8#	9#	10#
Plasma: CaCl_2_ (V: V)	1:0	499:1	498:2	497:3	496:4	495:5	494:6	493:7	492:8	491:9
Ca^2+^ concentration (mmol/L)	0	2	4	6	8	10	12	14	16	18
Degree of coagulation	−	−	−	+	++	++	++	++	++	++

One gram of frozen platelets was resuspended in 50 mL of 2_nd_ serum and subjected to three freeze–thaw cycles between −80 °C and 25 °C. The lysed mixture was then centrifuged at 6,000 × g for 30 min at 4 °C, and the supernatant was collected and passed through a 0.22 μm filter to obtain sterile PPL. Each batch of PPL was generated by pooling platelets from 12 donor pigs, and this process was repeated four times to produce four independent PPL/PFMG batches using the same donors and protocols. Preliminary characterization indicated that these batches exhibited similar material properties and biological activity. PFMG was then prepared by rapidly mixing porcine plasma, 10% PPL, and CaCl_2_ (to a final Ca^2+^ concentration of 8 mmol/L). The mixture was immediately dispensed into culture dishes or molds and allowed to clot, forming the 3D fibrin matrix gel.

### 2.3 Scanning electron microscopy

For scanning electron microscopy (SEM) sample preparation, PFMG was made at two different plasma concentrations (100% plasma and 75% plasma diluted with α-MEM), each with 3% PPL and 8 mmol/L CaCl_2_ added, and then allowed to clot in an incubator. After complete coagulation, the samples were fixed in methanol for 24 h and rinsed three times with phosphate-buffered saline (PBS). The gels were quickly frozen in liquid nitrogen for 10 min and then lyophilized for 24 h. Finally, the dried samples were sputter-coated with gold. The surface microstructure of each sample was observed under a SEM (Hitachi S-4800, Japan) to assess morphological differences.

### 2.4 Rheological measurements of PFMG

PFMG samples used for rheometry were prepared following the procedures for PFMG formation described above. Briefly, sterile porcine plasma was rapidly mixed with porcine platelet lysate (PPL) at a final ratio of approximately 3% and CaCl₂ was added to achieve a final Ca^2+^ concentration of ~8 mmol/L. The mixture was immediately dispensed into molds and allowed to clot undisturbed at 37 °C for 30 min to generate cohesive gels. For rheological testing, undiluted PFMG (100%) was used directly, and a half-strength formulation (50% PFMG) was obtained by diluting porcine plasma 1:1 with α-MEM; both preparations were analogous to those used for SEM analyses.

Dynamic oscillatory rheology was performed using an HR20 rheometer (TA Instruments, United States) equipped with a parallel-plate geometry. Gel disks were carefully transferred to the temperature-controlled stage, and excess material was trimmed. Measurements were carried out at 37 °C under a constant angular frequency of 6.28319 rad s^−1^ (1 Hz) with a small-amplitude oscillatory strain within the linear viscoelastic region, determined from preliminary strain-sweep experiments. Storage modulus (G′) and loss modulus (G″) were recorded as functions of time until the moduli reached steady values.

### 2.5 Isolation and culture of porcine mesenchymal stem cells

Mesenchymal stem cells (MSCs) were isolated from the bone marrow of three independent pigs following a previously described method (11) with modifications. Briefly, fresh pig ribs (healthy DLY female pigs, ~6 months old) were collected and transported on ice to the laboratory. Upon arrival, the samples were thoroughly disinfected by treatment with iodophor and 75% ethanol twice, followed by three rinses in cold PBS containing 5 × penicillin–streptomycin (P/S) for 10 min each. Muscle tissue was removed from the ribs, and the ribs were cut into small pieces using bone shears. The red bone marrow and cancellous bone from inside the rib pieces were scraped into a 100 mm culture dish containing 10 mL of culture medium. The following day, non-adherent debris was removed by changing the medium. Cells were expanded to 80–95% confluence and then cryopreserved. The growth medium for MSCs was α-MEM (Gibco) supplemented with 10% FBS (Sigma) or 8% PPL, 1% non-essential amino acids (NEAA; Gibco) and 1% GlutaMAX (Gibco).

To examine the suitability of different 3D PFMG configurations for MSC culture, we tested three culture modes: mixed culture (cells embedded within the PFMG), surface culture (cells seeded on top of a PFMG layer), and sandwich culture (cells seeded on PFMG and then overlaid with a second PFMG layer). Isolated MSCs were cultured in each of these PFMG configurations. Daily, phase-contrast microscopy was used to observe cell growth, morphology, and adhesion, including any changes after passaging.

### 2.6 Isolation and culture of porcine embryonic fibroblasts

Porcine embryonic fibroblasts (PEFs) were isolated using three independent embryos from a single sow according to a protocol for mouse embryonic fibroblasts (12, 13), with modifications for porcine tissue. In brief, uterine was collected from sows at 24.5 days of gestation immediately after slaughter. The abdomen was disinfected with 70% ethanol and the uterus was removed. Both ends of the uterus were tied with sterile cotton thread, and the uterus was excised and placed into a sterile bag for transport to the lab. In a biosafety cabinet, the uterus was thoroughly rinsed with sterile water and then sequentially scrubbed with hydrogen peroxide, iodophor, and 75% ethanol. After final sterile saline rinses, the uterus was opened to retrieve the embryos. The embryos were freed from extraembryonic membranes and washed in PBS several times until all maternal blood and debris were removed. The heads and visceral organs of the embryos were removed. The remaining tissue was minced with scissors. Ten milliliter of Type IV collagenase (Solarbio, China) were added to the minced tissue in a 15 mL tube, and the mixture was incubated at 37 °C for ~30 min, with gentle inversion every 5 min, until the suspension became viscous. The digest was centrifuged at 1,000 rpm for 5 min and the supernatant discarded. The pellet was washed with 5 mL of Dulbecco’s Modified Eagle Medium (DMEM) and incubated at 37 °C for 10 min, then centrifuged again, this wash step was repeated twice to remove residual enzymes. Finally, the cells and tissue fragments were resuspended in 12–15 mL of high-glucose DMEM (HyClone) supplemented with 10% FBS or 8% PPL, 1% NEAA, and 1% GlutaMAX, and transferred to a T75 flask. The culture was checked the following day, and cells that grew to 80–95% confluence were cryopreserved.

To explore whether different stereoscopic PFMGs can be used in cell culture, isolated PEFs were cultured in mixed, sandwich and surface cultures with PFMG. Photographs were taken under a microscope to observe the growth state of the PEFs daily and the morphological changes and adhesion conditions after passage.

### 2.7 Transcriptome sequencing and data analysis

Porcine mesenchymal stem cells (MSCs) were cultured in parallel on PFMG (3D, mixed format) or PPL (2D) conditions under 8% PPL conditions as described in cell culture part. After 96 h culture, total RNA was isolated from each sample using the TRIzol reagent (Invitrogen, United States) according to the manufacturer’s protocol. Three biological replicates were collected per condition (sample identifiers PPL_1124, PPL_1126 and PPL_1128 for the PPL group, and PFMG_1124, PFMG_1126 and PFMG_1128 for the PFMG group).

RNA quality was assessed with a Nanodrop 2000 spectrophotometer and Agilent 5300 Bioanalyzer to confirm that the RNA concentration exceeded 30 ng μL^−1^ and that the RNA Quality Number was above 6.5. Only samples with OD₂₆₀/₂₈₀ ratios between 1.8 and 2.2 and showing intact 18S and 28S rRNA bands on agarose gels were used for library preparation. Messenger RNA was purified from 1 μg of total RNA per sample using oligo-(dT) magnetic beads to capture polyadenylated transcripts. The enriched mRNA was fragmented to an average size of approximately 300 bp by incubation with fragmentation buffer, and first- and second-strand complementary DNA (cDNA) synthesis was performed using random hexamer primers and reverse transcriptase. Double-stranded cDNA fragments underwent end repair and 3′ adenylation, followed by ligation of sequencing adaptors. Size-selected fragments were amplified by polymerase chain reaction to generate the final libraries.

The cDNA libraries were sequenced using paired-end mode on a DNBSEQ-T7 platform (Meiji Bio, China). Raw reads were quality-filtered using fastp (Version 0.23.4) to obtain high-quality clean reads, which were then aligned to the *Sus scrofa* reference genome (Sscrofa11.1) with HISAT2 (version 2.2.1). Gene-level expression counts were generated and normalized as transcripts per million (TPM). Differential expression analysis between PFMG and PPL samples was conducted using DESeq2 (Version 1.42.0), with statistical significance defined by a Benjamini–Hochberg adjusted false-discovery rate < 0.05 and absolute log₂-fold change ≥ 1. Gene Ontology and Kyoto Encyclopedia of Genes and Genomes enrichment analyses were performed on the sets of differentially expressed genes using clusterProfiler. Gene Set Enrichment Analysis was also carried out using the GO terms annotated for *Sus scrofa* to identify over-represented biological processes. The raw sequencing data have been deposited in the NCBI Sequence Read Archive under BioProject ID: PRJNA1353812.

### 2.8 Cell growth curves

Third-passage PEFs and MSCs derived from three independent biological donors (n = 3 per cell type) were used to compare cell proliferation in different culture conditions. Cells were seeded in 6-well plates under three conditions: standard 2D culture with 15% FBS-supplemented medium, 2D culture with 10% PPL-supplemented medium, and 3D culture within 100% PFMG (with no additional serum in the medium). Each condition for each donor was seeded in triplicate wells with 6 × 10^4^ cells and cultured for 7 days. To construct growth curves, cells were harvested and counted every 24 h. For each donor, the average cell count from the three replicate wells at each time point was treated as a single data point. Growth curves were plotted by combining data from three independent experiments (i.e., three separate cell lines). For monolayer cultures (FBS or PPL conditions), cells were detached with 0.25% trypsin–EDTA (HyClone). For PFMG cultures, the fibrin gel was cut into small pieces and treated with a dedicated PFMG digestion solution (prepared as a 1:1 mixture of 0.05% w/v collagenase type I in DMEM and 0.05% w/v neutral protease in PBS) in a 37 °C water bath. The digestion mixture was incubated for 60 min with gentle mixing (inversion) every 10 min, then centrifuged at 190 × g for 15 min to collect the cells. Viability of recovered cells was assessed by Trypan Blue exclusion. The cells were resuspended in 1 mL of culture medium and counted using a Bürker counting chamber. Cell counts were recorded for each day, and proliferation curves were plotted for each culture condition.

### 2.9 Z-stack imaging

Fixed PFMG constructs were incubated in 4% paraformaldehyde (PFA) for 30 min at 4 °C to preserve cellular architecture. Constructs were incubated with DAPI solution for 10 min at RT protected from light. Z-stack images were acquired using an EVOS M5000 microscope system (invitrogen) with sequential channel scanning, resulting in a total stack thickness of 4 μm.

### 2.10 MSCs differentiation

To evaluate the multilineage differentiation potential of MSCs after culture in 3D PFMG, we first expanded MSCs under different PFMG conditions and then induced adipogenic, osteogenic, or chondrogenic differentiation. MSCs (~80% confluent) previously cultured in surface, sandwich, or mixed PFMG were harvested and re-seeded into 6-well plates maintained under the same respective PFMG condition at 6 × 10^4^ cells/well. Once cells in the surface PFMG group reached 80–90% confluence, all groups were switched to differentiation induction media (adipogenic, osteogenic, or chondrogenic, respectively), with medium changes every 2 days. The cells were maintained in the induction media for 21 days. For each of the three MSC donors, adipogenic, osteogenic and chondrogenic inductions were performed in duplicate wells per treatment group, and the entire induction series was conducted for all three donors to confirm reproducibility.

For adipogenic differentiation, the induction medium consisted of α-MEM with 10% FBS (or 8% PPL), 1% NEAA, 1% GlutaMAX, 100 nmol/L dexamethasone (Solarbio), 0.5 mmol/L IBMX (isobutylmethylxanthine; Solarbio), 200 μmol/L indomethacin (Solarbio), and 10 μmol/L insulin (Solarbio). For osteogenic differentiation, the medium was α-MEM with 10% FBS (or 8% PPL), 1% NEAA, 1% GlutaMAX, 100 nmol/L dexamethasone, 10 mmol/L β-glycerophosphate, and 50 μmol/L vitamin C (all from Solarbio). For chondrogenic differentiation, the medium was α-MEM with 10% FBS (or 8% PPL), 1% NEAA, 1% GlutaMAX, 100 nmol/L dexamethasone, 1 × insulin–transferrin–selenium (ITS; Sigma), 1% sodium pyruvate (Solarbio), and 50 μmol/L vitamin C.

### 2.11 Oil Red O staining (adipogenesis)

After 21 days of adipogenic induction, cells were stained with Oil Red O to confirm adipocyte formation. Briefly, the culture medium was removed, and cells were gently washed twice with PBS. Cells were fixed with 4% formaldehyde (Solarbio, G1262) for 20–30 min at room temperature. After fixation, cells were washed twice with distilled water and then rinsed with 60% isopropanol for 20–30 s. Freshly prepared Oil Red O working solution was added to each well and incubated for 10–20 min. Excess stain was then removed by rinsing with 60% isopropanol for 30 s and washing with distilled water multiple times until the background was clear. The lipid droplets retained the red stain. Nuclei were counterstained with Mayer’s hematoxylin for 1–2 min, followed by water rinses. Finally, an Oil Red O buffer was briefly added (1 min) and removed, and the cells were covered with distilled water for imaging. Stained cells were observed and photographed under a microscope.

### 2.12 Alcian Blue staining (chondrogenesis)

For verification of chondrogenic differentiation, cells were fixed and processed for Alcian Blue staining of sulfated glycosaminoglycans in the extracellular matrix. After removing culture medium, cell monolayers or cell-PFMG constructs were processed into paraffin sections. Slides were deparaffinized through graded ethanol (down to 95%) and air-dried. Sections were treated with acetic acid solution for 3 min to enhance stain uptake, then stained with Alcian Blue solution (Solarbio, G1560) for 30 min. After thorough rinsing in running water, Nuclear Fast Red solution was applied for 5 min to counterstain nuclei, followed by another water rinse. The slides were dehydrated through an ethanol series, cleared in xylene, and mounted with neutral resin. Chondrogenic matrix deposition (blue staining) was observed and imaged under a microscope.

### 2.13 Alizarin Red staining (osteogenesis)

To assess osteogenic differentiation, cells were fixed and stained with Alizarin Red S to detect mineralized calcium deposits. After induction, cultures were fixed, embedded in paraffin, and sectioned as above. Sections were deparaffinized in 95% ethanol and dried. Slides were then incubated in Alizarin Red S solution (Solarbio, G1450) for a few minutes, rinsed quickly with distilled water to remove excess stain, and counterstained if necessary. After thorough rinsing in water, sections were dehydrated, cleared with xylene, and mounted. The presence of red-orange mineralized nodules indicated osteogenic differentiation and was recorded microscopically.

### 2.14 Statistical analysis

Statistical analysis was performed using GraphPad Prism software (GraphPad Prism v9.5.0). Data are presented as mean ± standard deviation (SD). A two-way analysis of variance (ANOVA) was used to determine significance, followed by Dunnett’s multiple comparisons test. Statistical significance was defined as *p* ≤ 0.05.

## 3 Results

### 3.1 Development of standardized procedures to prepare PPLs and PFMGs

#### 3.1.1 Preparation of PPL

**Figure 1:**
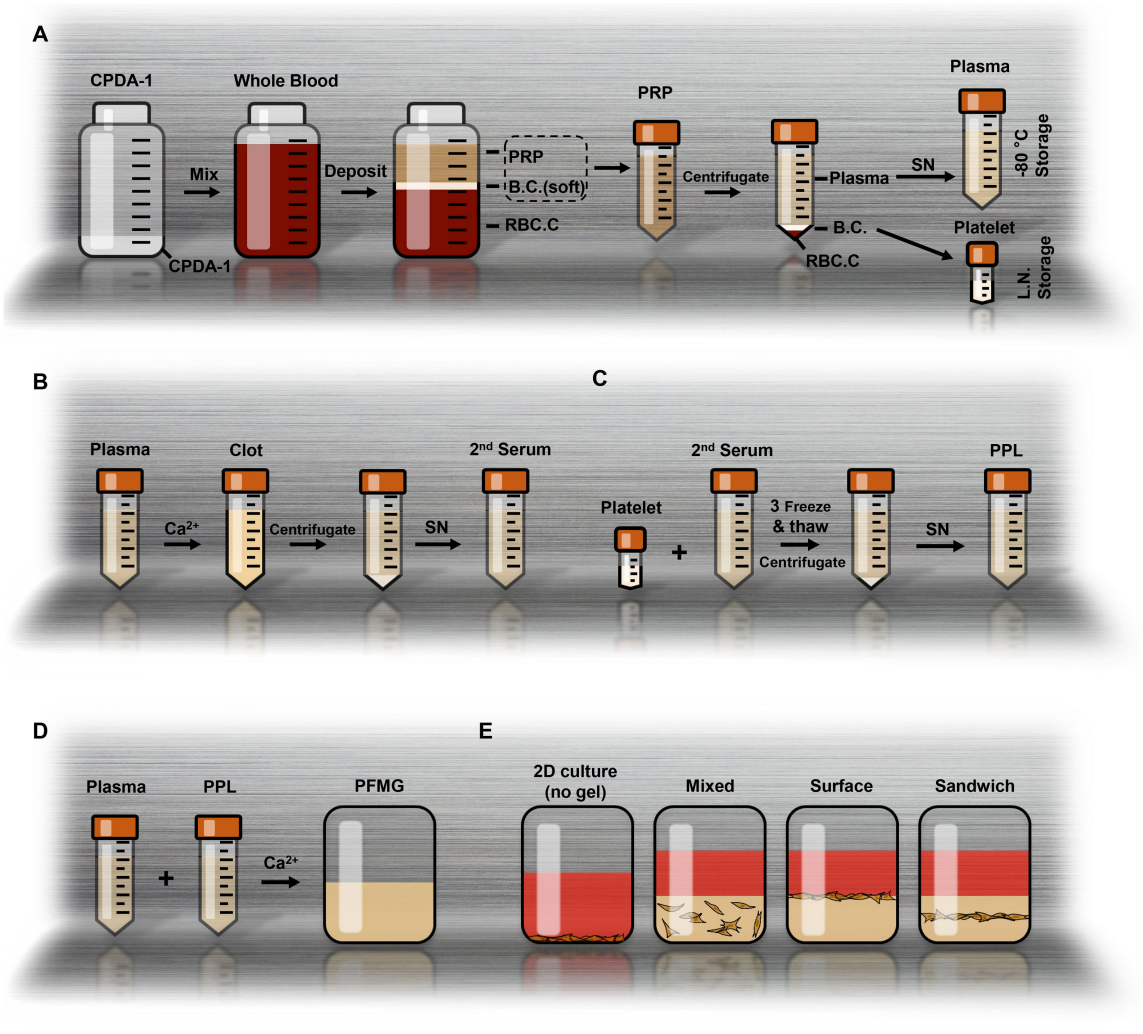
Preparation of PPL and PFMG for cell culture. **(A)** Schematic illustrating the separation of whole blood into platelet-rich plasma (PRP), buffy coat (B.C.), and red blood cell concentrate (RBC.C) using CPDA-1. **(B)** Preparation of second serum (2nd Serum) by adding Ca^2+^ to plasma and inducing clotting, followed by centrifugation. **(C)** Resuspension of platelet concentrate with 2nd serum, followed by freeze–thaw cycles and centrifugation to obtain PPL. **(D)** Composition of PFMG: mixing plasma, PPL, and Ca^2+^, followed by coagulation to form PFMG. **(E)** Schematic showing different cell culture methods employing PFMG: including mixed culture; surface culture; and sandwich culture.

We first optimized the method for preparing platelet lysate from porcine blood, adapting techniques from human PL protocols (14). In typical human PL preparation, PRP is separated from anticoagulated whole blood and platelets are lysed by repeated freeze–thaw cycles or ultrasonication, with anticoagulants added throughout to prevent clotting. Similarly, for PPL production in our study, whole pig blood (~1 L) was collected into a container pre-coated with 123 mL of CPDA-1 anticoagulant. After standing at 20–22 °C for 1 h, the blood separated into three layers: the upper PRP layer, a middle buffy coat rich in platelets (with some leukocytes), and a lower red blood cell layer. PRP was carefully drawn off just above the buffy coat and transferred to centrifuge tubes. Three thousand and two hundred rpm centrifugation for 20 min pelleted the platelets, separating them from the plasma. The plasma supernatant was either stored at −80 °C or processed further to produce serum. The platelet pellet (buffy coat) was isolated from any residual red cells (if significant red cell volume was present, the red cell portion was removed by trimming the tube) and then distributed into sterile cryovials. These platelet concentrates were weighed, labeled, and cryopreserved in liquid nitrogen (Figure 1A).

To generate fibrinogen-depleted serum for platelet lysis, a small fraction of platelets along with CaCl_2_ were added back to the anticoagulated plasma. The reintroduction of free Ca^2+^ initiated coagulation, and incubation at 37 °C for ~30 min converted the plasma into a fibrin clot. After clot formation and centrifugation, the clear supernatant (fibrinogen-depleted porcine serum) was collected as the second-regenerated serum (2nd serum) (Figure 1B). The cryopreserved platelets were then resuspended in the 2nd serum at a ratio of 1:50 (mass of platelets: volume of serum). The suspension was subjected to three freeze–thaw cycles and centrifuged to remove cellular debris and platelet membranes. The resulting supernatant was filtered (0.22 μm) to yield the PPL (Figure 1C).

#### 3.1.2 Preparation of PFMG

During the production of the 2nd serum, we noticed that the coagulated plasma clots had a gel-like consistency, suggesting a potential use as a 3D culture matrix. To create a sterile version of this fibrin matrix, the frozen plasma was melted and passed through a 0.22 μm filter. We then established a protocol to formulate PFMG by combining filtered porcine plasma with 10% PPL and an appropriate amount of CaCl_2_ (final Ca^2+^ ~ 8 mmol/L). This mixture was quickly pipetted or inverted to mix and then dispensed into culture dishes, where it rapidly coagulated into a solid gel (Figure 1D).

Using this approach, we ried three distinct 3D culture methods to test the effects of PFMG on cell behavior: (1) mixed culture—cells were mixed directly into liquid plasma/PPL before gelation, embedding them within the matrix; (2) surface culture—a layer of PFMG was allowed to solidify in the dish, and cells were then seeded on top; and (3) sandwich culture—cells were first seeded on a solidified PFMG layer, allowed to attach for ~12 h, then covered with a second layer of PFMG (Figure 1E). We also empirically determined optimal volumes of PFMG for various plate sizes to achieve roughly 1–2 mm gel thickness (Table 2).

**Table 2:** Optimum volume of PFMG prepared in different petri dishes.

Cell culture plates	PFMG volume
60 mm dish	2 mL
35 mm dish	1 mL
6-well plates	1 mL
12-well plates	0.5 mL
24-well plates	0.3 mL
96-well plates	0.05 mL

### 3.2 Characterization of PFMG properties: scaffold functionality and molecular sieving

#### 3.2.1 Scaffold function of PFMG

**Figure 2:**
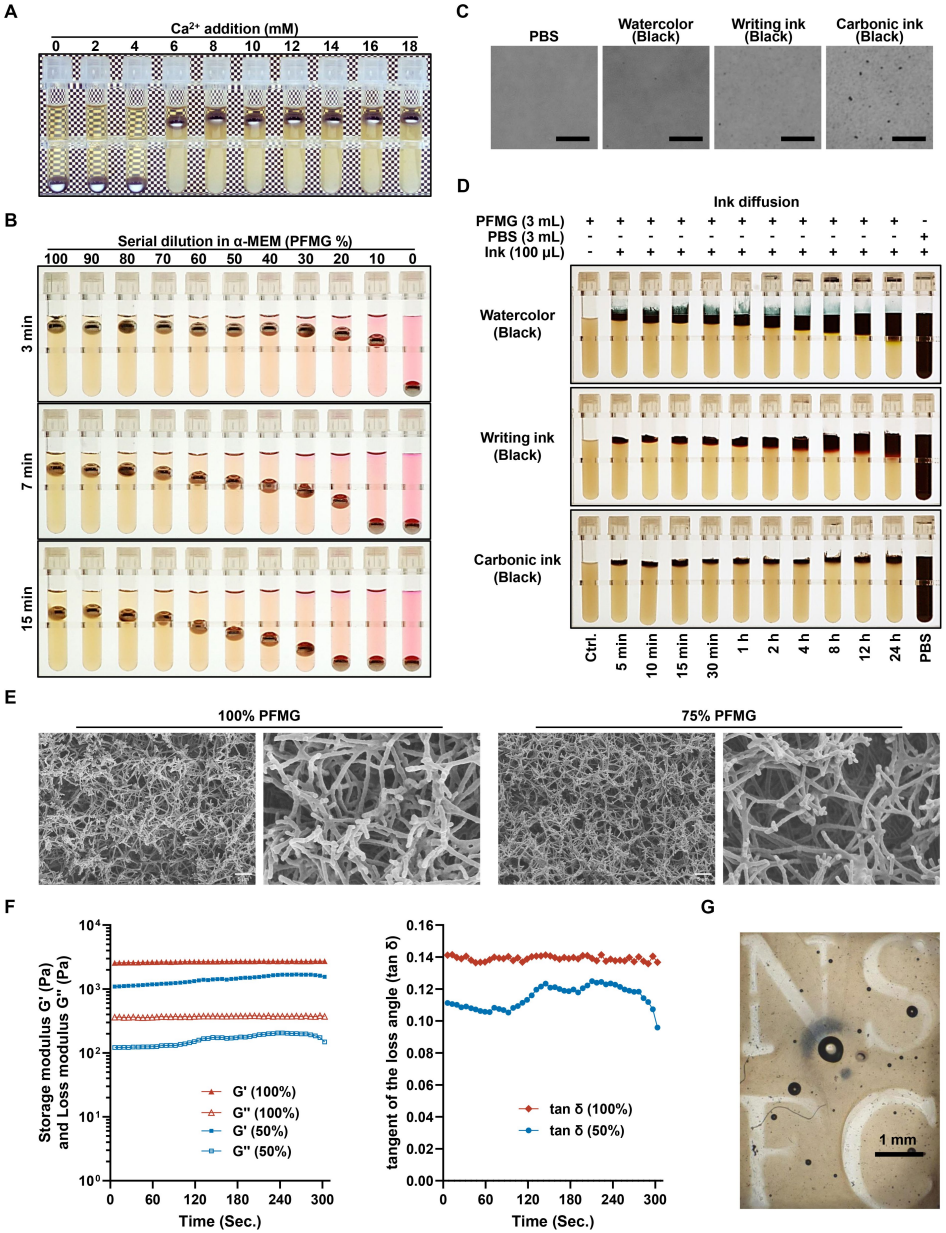
Characterization of PFMG properties and functional analysis. **(A)** Stable coagulation of plasma with varying concentrations of Ca^2+^ (0–18 mmol/L). **(B)** Metastable coagulation of plasma using low level of Ca^2+^, and PFMG was serially diluted (100% PFMG diluted to 0% PFMG) in α-MEM, and recording the displacement of the steel balls at 3 min, 7 min, and 15 min. **(C)** Particles observation of different types of ink (watercolor, writing ink, and carbonic ink) in PBS, observed under a microscope (scale bars: 10 μm). **(D)** Comparison of the penetration depth of watercolor ink, writing ink, and carbonic ink in PFMG and PBS over 24 h. **(E)** Microstructural images of 100% PFMG and 75% PFMG, observed under a scanning electron microscope (SEM). **(F)** Rheological measurements of G′ (storage modulus) and G″ (loss modulus) for 100 and 50% PFMG over time using a rheometer. The 100% PFMG exhibited higher modulus values. **(G)** Tan δ (the ratio of G″ to G′) values for 100 and 50% PFMG over time, showing that 100% PFMG is more stable. **(H)** Micrographs of PFMG gel shaped using movable type (9# type) molds, demonstrating the plastic potential of PFMG. The letters “NSFC” are included as a tribute to the National Natural Science Foundation of China.

Excessive calcium in cell culture systems can perturb cell membranes (15) and precipitate factors that adversely affect cells. To minimize such adverse effects, we sought to identify the lowest CaCl₂ concentration sufficient for fibrinogen coagulation when preparing the 2nd serum and PFMG. We titrated anticoagulated plasma with increasing amounts of CaCl₂ and assessed clot integrity by placing a 9 mm steel ball on each gel after 30 min of coagulation. Final Ca^2+^ concentrations below 6 mmol/L yielded incompletely polymerized matrices in which the steel ball sank to the bottom. Stable fibrin clots that resisted compression were consistently obtained at Ca^2+^ concentrations of 8 mmol/L and above (Figure 2A). While for a further elevation of Ca^2+^ produced a counter-intuitive effect: at high concentrations (≥80 mmol/L), coagulation was impaired and a cohesive gel failed to form.

Interestingly, at a lower level of Ca^2+^ supplement (~5.5 mmol/L), with longer coagulation (over 1 h), the resulting gels became noticeably softer, and the steel ball gradually compressed the matrix. To further evaluate this phenomenon, we prepared PFMG diluted with α-MEM to various levels and coagulated with 10% PPL and 5.5 mmol/L CaCl₂ for 2 h at 37 °C. When steel balls were placed on these gels, undiluted gels (80–100% PFMG) compressed slightly under the weight of the ball, whereas 30–70% dilutions showed progressively greater compress, 10–20% gels collapsing substantially within 7–15 min (Figure 2B). This ability to tailor PFMG stiffness by modulating PPL concentration suggests potential utility for specialized applications requiring softer scaffolds.

The moldability of PFMG was demonstrated by shaping gels in movable-type molds to form letters and symbols (Figure 2G). The gel reproduced fine details of the molds-illustrated by the clear “NSFC” characters, showing that PFMG can be cast into customized 3D constructs. Together with the observation that reduced PPL concentrations yield pliable gels, this plasticity suggests that PFMG could be adapted as a versatile scaffold for tissue repair patches, organ engineering, or other 3D culture applications requiring precise geometry and tailored mechanical properties.

#### 3.2.2 Molecular sieve characteristics of PFMG

To further examine the permeability and molecular sieving properties of PFMG, we performed a simple diffusion assay using inks containing particles of different sizes. Three types of ink were used: watercolor ink (soluble dye, small molecules), writing ink (fine carbon particles and soluble dye), and carbon ink (larger carbon particles). Each ink was diluted and also smeared on a glass slide for particle size observation (Figure 2C): PBS showed a clear background with no particles, watercolor ink appeared homogeneous with no visible particulates (only soluble color), writing ink showed fine dispersed carbon particles, and carbon ink had clearly larger particulate matter. Next, 3 mL PFMG gels were prepared in a series of tubes, and 100 μL of each ink was applied on top of the gels. The inks were added in staggered time intervals so that at the end of the experiment, each ink had diffused for a known duration before images were taken.

The diffusion results (Figure 2D) revealed that small molecules diffuse readily through PFMG, whereas larger particles are impeded. Watercolor ink (small dye molecules) permeated deeply: after 10 min it reached ~5 mm into the gel, and after 24 h it had diffused ~2 cm. Writing ink (with fine particles) had slightly slower diffusion: ~5 mm in 2 h and ~1.3 cm in 24 h. In contrast, the carbon ink (large particles) showed only minimal shallow penetration even after 24 h. These observations suggest that PFMG allows rapid diffusion of small nutrients and gasses (e.g., oxygen, glucose, etc.), like loose-connective tissue stroma, but effectively traps or slows larger macromolecules. In other words, PFMG behaves as a molecular sieve, permitting efficient exchange of small molecules while restricting the movement of large complexes, which could be beneficial for maintaining localized trophic or signal factors around cells.

#### 3.2.3 Microstructure of PFMG scaffold

We next observed the microarchitecture of PFMG by SEM. PFMG samples were prepared with 100 and 75% plasma (diluted in α-MEM), then fixed and processed as described in Section 2.3. SEM imaging (Figure 2E) showed that PFMG forms a typical branched fibrillar network. The fibrin fibers created an interwoven scaffold resembling an extracellular matrix structure. In the 75% plasma sample, the network appeared less dense (thinner fibers and larger pores) compared to the undiluted plasma PFMG, consistent with fewer fibrinogen molecules available for polymerization. These images confirm that PFMG can serve as a 3D fibrous scaffold analogous to connective tissue stroma, and the density of its network can be adjusted by altering plasma concentration. The SEM findings align with our diffusion experiments, indicating that PFMG’s porous structure allows it to act as a supportive scaffold with molecular sieve characteristics. Collectively, these data suggest that PFMG can effectively simulate an *in vivo*-like interstitial environment for cell culture.

#### 3.2.4 Rheological properties of PFMG

In addition to microstructural observations, we quantified the viscoelastic properties of PFMG (Figures 2F,G). Dynamic rheology was performed under controlled conditions (6.28319 rad/s, 37 °C) using an HR20 rheometer, allowing direct comparison of undiluted (100%) and half-strength (50%) PFMG. The storage modulus (G′) of 100% PFMG averaged 2673.09 ± 47.75 Pa, whereas 50% PFMG exhibited a reduced G′ of 1406.41 ± 200.23 Pa. The corresponding loss moduli (G″) were 371.64 ± 1.15 Pa for 100% PFMG and 162.29 ± 29.94 Pa for the 50% dilution. These measurements (Figure 2F) demonstrate that undiluted PFMG is markedly stiffer and dissipates more energy than the 50% dilution, which aligns with our observation that higher plasma concentrations produce more robust fibrin networks. The tan *δ* (G″/G′) ratios were higher for 100% PFMG, indicating a stiffer gel with higher viscous character, whereas 50% PFMG displayed a lower tan δ, reflecting its softer, but much elastic character (Figure 2G). Overall, the rheological data provide quantitative confirmation that PFMG offers tunable mechanical properties that could influence cell behavior in 3D culture.

### 3.3 PFMG promotes porcine cell proliferation

**Figure 3:**
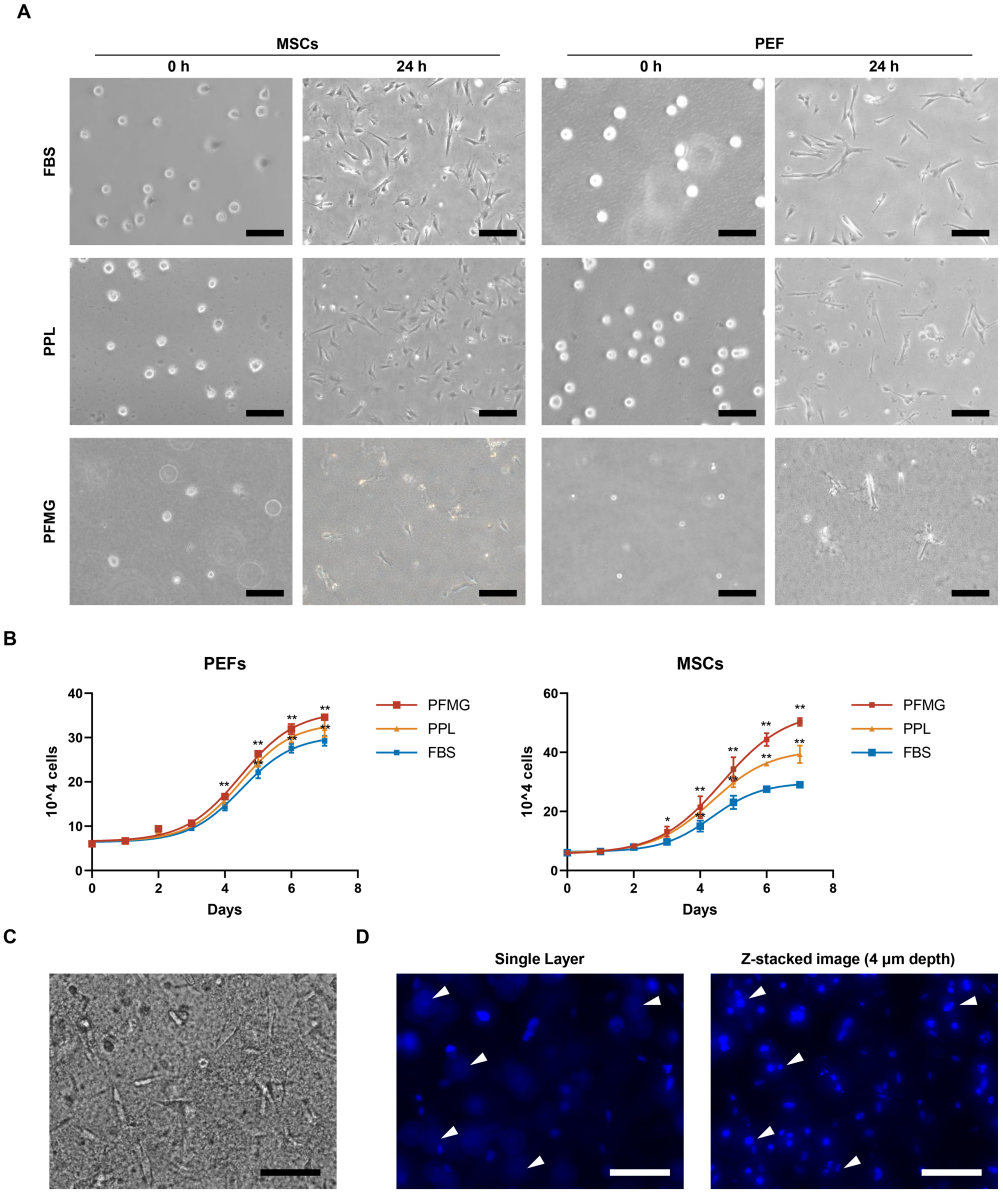
Cell growth characteristics of PFMG. **(A)** MSCs and PEFs were cultured in FBS, PPL, or PFMG for 0 h or 24 h. Photographs were taken to assess cell morphology and attachment. Scale bars: 250 μm. **(B)** Growth curves of MSCs and PEFs under different culture conditions: comparison of PFMG (mixed culture) and PPL with FBS as control. Data were acquired from 3 independent experiments (*p ≤ 0.05, **p ≤ 0.01). **(C)** MSCs cultured in PFMG exhibited a 3D distribution across different layers of the matrix, resulting in relatively few cells being visible in single focal planes under the microscope. **(D)** After DAPI staining of nuclei and subsequent Z-stack imaging (4 μm in), cell nuclei were clearly observed at multiple depths within the PFMG. The tips of the white arrows indicate nuclei that are out of focus within a single optical plane. Scale bars: 250 μm.

We next compared porcine cell growth in the 3D PFMG matrix (mixed format) with that in conventional two-dimensional (2D) cultures. Initially, MSCs and PEFs seeded in FBS-, PPL- and PFMG-based (mixed format) systems were spherical, after 24 h, cells on FBS- or PPL-supplemented plastic adhered and adopted a spindle-shaped morphology, whereas those embedded in PFMG developed star-like shapes with multiple projections typical of 3D growth (Figure 3A). A detailed statistical analysis of the growth curves was performed on the direct cell count data collected over 7 days. For PEFs, the proliferation rate in PFMG was not statistically changed. On day 4, proliferation in PFMG became significantly higher compared to FBS (*p* < 0.05). From days 5 to 7, PFMG-supported PEF growth was significantly higher than both FBS and PPL (*p* < 0.05). A similar trend was observed for MSCs. By day 3, MSCs proliferation in PFMG was significantly higher than in FBS (*p* < 0.05), while the difference compared to PPL remained non-significant. From days 4 to 7, the proliferation of MSCs in PFMG was significantly greater than in both FBS- and PPL-supplemented 2D cultures (*p* < 0.05). Consequently, PFMG cultures ultimately yielded markedly higher total cell numbers (Figure 3B). These observations were corroborated by Z-stack imaging of DAPI-stained MSCs, nuclei were distributed throughout the depth of the gel and were far more numerous than could be appreciated in a single focal plane (Figures 3C,D). Collectively, the morphological and quantitative data indicate that the nutrient-rich fibrin network and 3D architecture of PFMG provide a more supportive environment for porcine cell expansion than traditional 2D cultures.

### 3.4 PFMG supports multilineage differentiation of MSCs

**Figure 4:**
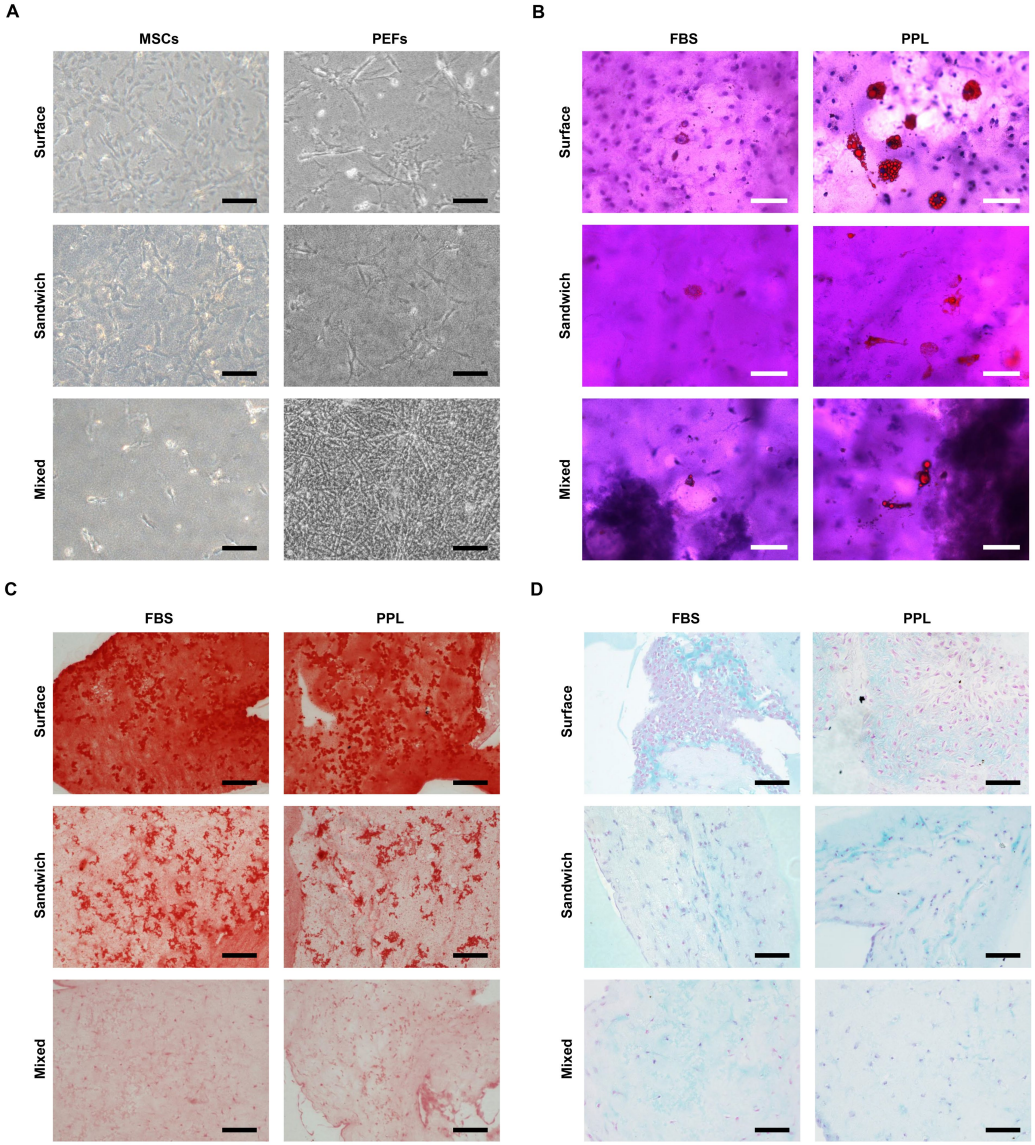
Multilineage differentiation potential of MSCs cultured in PFMG. **(A)** Surface, sandwich, and mixed cultures of MSCs and PEFs in PFMG. Scale bars: 250 μm. **(B)** Surface, sandwich, and mixed cultures of MSCs in PFMG for adipogenic differentiation with FBS and PPL induction media. Scale bars: 100 μm. **(C)** Surface, sandwich, and mixed cultures of MSCs in PFMG for osteogenic differentiation with FBS and PPL induction media. Scale bars: 200 μm. **(D)** Surface, sandwich, and mixed cultures of MSCs in PFMG for chondrogenic differentiation with FBS and PPL induction media. Scale bars: 100 μm.

To determine whether 3D culture alters lineage potential, porcine MSCs were embedded in PFMG in three formats (surface, sandwich and mixed culture) and subjected to adipogenic, osteogenic and chondrogenic induction. In all configurations, cells remained viable and exhibited fusiform or star-like morphologies consistent with healthy 3D growth (Figure 4A). After 3 weeks of induction, staining with Oil Red O, Alizarin Red and Alcian Blue confirmed that adipocytes, osteoblasts and chondrocytes had formed in each PFMG culture mode, demonstrating retention of multipotency (Figures 4B–D). Replacing FBS with PPL in the induction media did not affect osteogenic or chondrogenic outcomes but yielded more abundant lipid accumulation during adipogenesis (Figure 4B). These findings indicate that PFMG provides a supportive 3D niche that preserves the multilineage differentiation capability of MSCs and that porcine platelet lysate can substitute for FBS in porcine differentiation protocols while promoting adipogenesis.

### 3.5 Transcriptomic results and analysis

**Figure 5:**
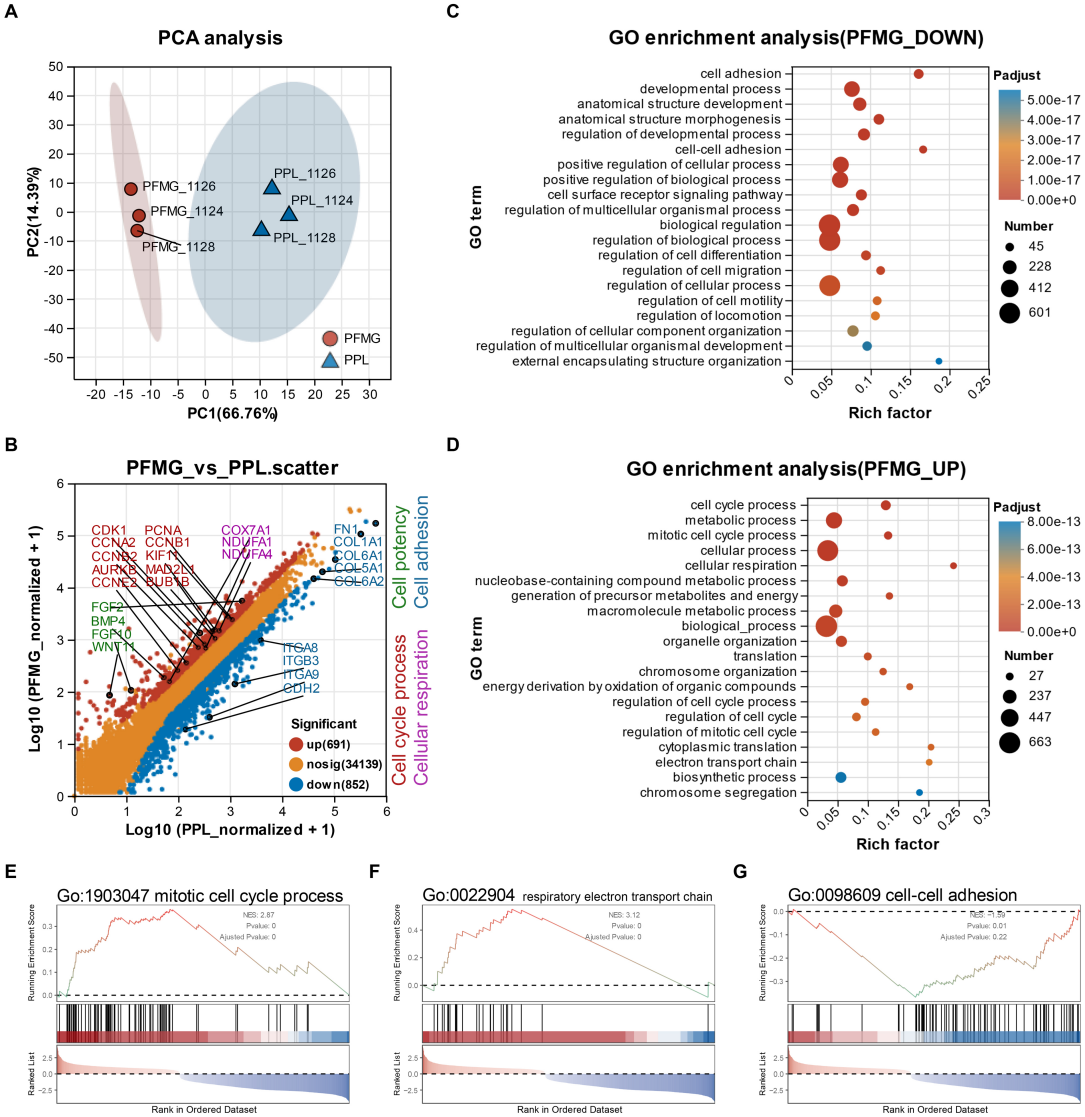
Transcriptome analysis of MSCs cultured in PFMG vs. PPL (2D vs. 3D). **(A)** Principal component analysis (PCA) showing clustering of MSCs from three different individuals cultured in PPL and PFMG. The data indicate good reproducibility in gene expression levels across individual samples, with distinct differences between 2D and 3D culture conditions. **(B)** Differential gene expression scatter plot highlighting 691 upregulated genes and 852 downregulated genes in PFMG culture conditions. Genes related to cell cycle process, cellular respiration, and cell potency are upregulated in PFMG, while genes associated with cell adhesion are upregulated in PPL culture conditions. **(C)** Gene ontology (GO) enrichment analysis for genes upregulated in PFMG (PFMG_UP) showing biological processes related to mitotic cell cycle regulation, chromosome segregation, and cellular respiration. **(D)** GO enrichment analysis for genes downregulated in PFMG (PFMG_DOWN) highlighting biological processes such as regulation of cell migration, and cell–cell adhesion. **(E)** Gene set enrichment analysis (GSEA) for the GO term “mitotic cell cycle process” (GO:1903047), and **(F)** “respiratory electron transport chain” (GO:0022904), demonstrating increased activity in PFMG cultures, **(G)** GO term “cell–cell adhesion” (GO:0098609), highlighting differences in the role of PFMG and PPL in cellular processes.

To explore the molecular basis of the phenotypic change observed and other changes beyond observation in PFMG cultures, we performed bulk RNA sequencing on MSCs grown in 3D PFMG and compared them with cells cultured in 2D PPL condition. Principal component analysis (PCA) of the transcriptomic data revealed tight clustering of biological replicates and clear segregation between PFMG and PPL samples along the first principal component, indicating that the 3D matrix induces a distinct transcriptional program (Figure 5A). Differential expression analysis identified 1,543 genes with significant changes between conditions (691 upregulated and 852 downregulated in PFMG compared with PPL). The differential gene expression scatter plot in Figure 5B illustrates transcriptional changes and highlights genes highly associated with cell cycle and mitotic processes (CDK1, PCNA, CCNA2, CCNB1, CCNB2, CCNE2, etc.), cellular respiration (COX7A1, NDUFA1, NDUFA4, etc.) and cell potency related genes (FGF2, BMP4, FGF10, WNT11, etc.) were predominantly upregulated in the PFMG group. In contrast, genes involved in cell–cell adhesion and extracellular matrix (CDH2, COL1A1, COL6A1, FN1, ITGA8, etc.) were more highly expressed in PPL-cultured cells.

Gene Ontology enrichment analysis further underscored these trends. Upregulated genes in PFMG cultures were enriched for biological processes related to mitotic cell cycle regulation, chromosome segregation and oxidative phosphorylation (Figure 5C). Downregulated genes were enriched in terms associated with regulation of cell migration and cell–cell adhesion (Figure 5D). Gene set enrichment analysis (GSEA) corroborated these findings: the gene sets “mitotic cell cycle process” (GO:1903047) and “respiratory electron transport chain” (GO:0022904) were significantly enriched in the PFMG group, whereas the “cell–cell adhesion” (GO:0098609) set was enriched in the PPL group (Figure 5D). Collectively, these data indicate that the 3D PFMG matrix promotes transcriptional program that favor proliferation and metabolic activity while suppressing adhesion-related pathways.

These molecular shifts align with our phenotypic observations. The enhanced expression of cell cycle and respiratory genes correlates with the markedly higher proliferation of MSCs and fibroblasts cultured in PFMG compared with those grown in 2D media. The downregulation of adhesion genes likely reflects the reduced substrate dependence of cells embedded within the 3D fibrin network, consistent with their 3D distribution and diminished spreading observed in microscopy (Figures 3C,D). Overall, the transcriptome analysis suggests that PFMG not only provides mechanical and biochemical cues that enhance cell growth but also reprogrammes MSCs toward a proliferative and metabolically active state, thereby underpinning the improved expansion and functionality observed in the 3D scaffold.

## 4 Discussion

In this study, we set out to build a fully porcine, growth-factor–rich 3D culture system that simultaneously overcomes the inherent technical trade-off between media stability and scaffold utility in platelet-derived systems. By cleanly decoupling plasma and platelet fractions through a standardized workflow, using a controlled replenishment step to convert fibrinogen into a fibrin matrix, and then preparing a heparin-free PPL in fibrinogen-depleted 2nd serum, we effectively turned fibrinogen from a clotting nuisance during PL employed cell culture into a useful biomaterial. The resulting PFMG showed tunable mechanical properties and molecular sieving behavior, and it markedly enhanced the proliferation of MSCs and PEFs while preserving the tri-lineage differentiation capacity of MSCs. The core innovation of our approach lies in the establishment of a synergistic “matrix + lysate” ecosystem via a “separation-and-reintegration” strategy, which ensures precise spatiotemporal control over gelation while maintaining a pure, potent biochemical environment free from heparin interference. This fully species-matched platform directly addresses key practical challenges in PL application without circumventing the use of whole blood. These findings position PFMG not only as a technical solution to long-standing PL preparation issues, but also as a versatile and sustainable platform for species-matched 3D culture in livestock and translational research.

At the same time, our results confirm and extend several themes emerging from the fibrin and PL literature. Fibrin hydrogels are known to offer excellent cytocompatibility and tunable stiffness that can be adjusted through fibrinogen and Ca^2+^ concentrations, making them attractive for 3D cell encapsulation and delivery (16–18). Likewise, human PL has been repeatedly shown to support robust expansion of MSCs and other cell types and is increasingly viewed as a viable substitute for fetal bovine serum (FBS) (19–21). By integrating these two concepts in a species-specific manner, we created a porcine system in which fibrin provides the 3D architecture and PPL supplies a built-in reservoir of growth factors. This combination allowed us to culture porcine cells in a xeno-free, physiologically relevant environment while using blood that is inexpensive and readily available as a by-product of the pork industry (22, 23).

### 4.1 Standardized preparation of porcine platelet lysate

When we started this work, we were particularly concerned with two well-recognized problems in PL preparation: spontaneous fibrin clot formation due to residual fibrinogen, which causes medium gelling and cell aggregation, and the need to add heparin to prevent clotting, which introduces new sources of variability and potential cytotoxicity. Human PL typically contains substantial amounts of fibrinogen and coagulation factors, this can lead to visible gels and precipitates during storage and in cell culture, even when heparin is present (14, 24). High heparin concentrations, however, can inhibit proliferation and induce apoptosis in several cell types, and have been reported to alter MSC phenotypes *in vitro* (19, 25). Recent efforts therefore focus on depleting fibrinogen—either mechanically or via controlled clotting—to obtain fibrinogen-depleted PL that can be used without heparin while still supporting MSCs expansion (14, 26). In parallel, several groups have shown that fibrin clots tend to adsorb and retain growth factors, which might be beneficial in a scaffold but is problematic when the goal is to keep cytokines in soluble form for use as a medium supplement (17, 18).

Our workflow was designed explicitly around these issues. Instead of lysing platelets directly in plasma, we first separated plasma and platelets, then induced a controlled coagulation step by adding titrated Ca^2+^ and a small amount of PL to the anticoagulated plasma. This converted fibrinogen into a fibrin clot that we repurposed as the PFMG scaffold, while the centrifugally harvested supernatant became a fibrinogen-depleted “second serum” used to prepare PPL. This approach is conceptually similar to recently described CaCl₂-based protocols for fibrinogen-depleted human PL, which also support heparin-free MSC expansion, but we extend it by explicitly exploiting the clot as a 3D matrix rather than discarding it (14, 26, 27). In our hands, the final PPL remained non-clotting in culture, and we observed no need for heparin, while growth promotion of porcine cells was at least comparable, and often superior, to FBS-containing media, in line with reports that PL can replace FBS without compromising MSCs proliferation or differentiation (19, 21, 28). By converting a source of clotting problems (plasma fibrinogen) into a useful biomaterial (PFMG) and using the fibrinogen-depleted phase to generate stable PPL, we addressed both medium gelling and growth-factor adsorption within a single integrated system.

### 4.2 Basic properties and functional features of PFMG

PFMG formation relies on controlled reintroduction of calcium to fibrinogen-depleted plasma supplemented with a defined fraction of platelet lysate. The Ca^2+^ cross-linking triggers polymerization of fibrin monomers to form a stable 3D network (17, 29). Our titration experiments showed that a final Ca^2+^ concentration of 8 mmol/L consistently produced a mechanically robust gel, whereas lower concentrations led to weak or incomplete clotting. This behavior is consistent with the broader fibrin literature, where Ca^2+^-dependent cross-linking are key determinants of stiffness and network integrity (17, 30). To further tune the gel’s mechanical properties, we diluted the plasma to 75% produced thinner fibrin fibers and larger pores (observed by scanning electron microscopy), corresponding to a softer, more compliant matrix. Our system achieves this tunability with minimal reagents, relying on endogenous porcine plasma proteins, CaCl₂ and platelet lysate, rather than complex engineered composites.

Functionally, PFMG acts as a size-selective molecular sieve. In our diffusion assay, small dye molecules rapidly permeated the 1–2 mm thick gel, whereas larger particulates were retarded, demonstrating size-dependent transport through the fibrin network. Taken together, PFMG offers a simple, tunable, fully porcine matrix that integrates biochemical (platelet-derived signals) and biophysical (3D fibrin) cues to support cell growth.

### 4.3 Biological effects of PFMG on cell proliferation and *in vitro* development

Biologically, our data show that PFMG provides a highly supportive niche for porcine cells. Both MSCs and PEFs initially displayed a short adaptation phase in the 3D gel, but from day 4 onward their growth curves in PFMG surpassed those in 2D cultures with either FBS or PPL supplementation. This acceleration is consistent with reports that PL outperforms FBS for MSCs expansion in multiple species, including humans, dogs and other large animals (19, 28, 31). In our case, the advantage is likely amplified by the 3D fibrin architecture: embedded cells adopt star-like morphologies, establish multicellular networks throughout the gel, and interact intimately with the matrix, as seen in Z-stack imaging. Similar morphology-dependent enhancements of proliferation and matrix production have been described for fibroblasts and MSCs in other 3D hydrogels, where spatial freedom and matrix contact potentiate growth factor signaling (32–34). Given that platelets release potent mitogens such as PDGF, TGF-β, and IGF-1, which drive MSCs cycling and survival (23, 35, 36), we interpret PFMG as a “self-feeding” matrix in which biophysical and biochemical cues are tightly integrated: cells sense the 3D fibrin network while simultaneously being surrounded by platelet-derived factors that continuously diffuse from the gel.

Importantly, we did not observe any loss of stemness in PMSCs after culture in PFMG. Cells expanded in PFMG retained robust tri-lineage differentiation into adipogenic, osteogenic and chondrogenic lineages, comparable to standard 2D cultures. It is noteworthy, however, that while MSCs under all three PFMG configurations (surface, sandwich, and mixed) successfully differentiated into adipocytes, osteocytes, and chondrocytes, the efficiency was relatively weaker in the mixed group. This attenuated differentiation may be attributed to restricted diffusion of induction factors within the gel and greater physical confinement of the cells in the mixed configuration, which could collectively impair the transmission of differentiation signals. For instance, adipogenesis typically requires substantial lipid accumulation and cell rounding, processes that may be hindered within a dense 3D network. Furthermore, the fully embedded condition may limit certain cell-matrix and cell–cell interactions that are conducive to differentiation—particularly for osteogenesis, where cell spreading on a stiffer surface facilitates induction, a condition potentially difficult to achieve when cells are completely enveloped. Critically, the gene expression changes observed in PFMG-cultured cells are attributable to the 3D culture environment itself, rather than to differences in medium composition, as both the PFMG and the control 2D conditions were supplemented with PPL. In fact, adipogenic differentiation was notably stronger when induction was performed in the presence of PPL, suggesting that platelet-derived signals may bias MSCs toward adipogenesis under appropriate hormonal cues. This agrees with studies showing that PL can enhance or modulate adipogenic and osteogenic differentiation without compromising overall multipotency (37–39). Our preliminary transcriptomic analysis further supports this view: MSCs grown in PFMG showed up-regulation of genes related to cell-cycle progression and growth-factor signaling, while maintaining expression of key MSCs markers. These trends mirror observations in MSCs cultured with PL or in fibrin-rich niches, where proliferation-associated genes and paracrine factors are elevated but differentiation capacity remains intact (20, 40, 41). Taken together, we regard PFMG as a niche that not only accelerates porcine cell expansion but also preserves, and in some contexts even potentiates, their developmental plasticity.

### 4.4 Prospects for tissue engineering and regenerative biology

From an application perspective, we see several advantages of PFMG for tissue engineering and regenerative biology. First, PFMG is scalable and cost-effective: pig blood is abundant in agricultural settings, and our workflow uses only standard laboratory reagents (CPDA-1, CaCl₂) without expensive recombinant proteins. This aligns with the broader move toward using PRF/PRP and fibrin-based products as accessible biomaterials that support cell adhesion, viability and differentiation in various tissue models (33, 34, 42). Second, the material is highly configurable. By adjusting plasma concentration, Ca^2+^ levels or blending with other polymers, we can tune stiffness and degradation to match specific tissues, much as has been done in engineered fibrin hydrogels for bone, cartilage and muscle regeneration (16, 18, 43). Third, because PFMG and PPL are species-matched, our approach can be extended to other key livestock or preclinical species (e.g., bovine, ovine, canine), enabling xeno-reduced, or even xeno-free culture systems tailored to particular animal models or organ-donor candidates such as pigs for xenotransplantation research (22, 28).

Looking ahead, we envision PFMG being used in several directions. As a 3D culture matrix, it can serve as a platform to build porcine organoid or tissue-like constructs that better recapitulate *in vivo* microenvironments than rigid plastic or purely synthetic gels. As a cell-delivery vehicle, PFMG could be combined with porcine MSCs or other progenitors to explore regenerative processes in preclinical models, analogous to how PRF and fibrin sealants have been used to enhance bone and soft-tissue repair (36, 44). Finally, because our workflow cleanly separates and re-integrates plasma and platelet fractions, it offers a general blueprint for generating paired “matrix + lysate” systems in different species-each providing a self-derived 3D scaffold and a serum substitute optimized for that species. This combination of practicality, tunability and biological relevance is what makes PFMG particularly attractive as a foundation for future work in 3D cell culture and regenerative biology.

### 4.5 Limitations and future directions

Although PFMG demonstrates significant advantages over traditional serum-based systems, several limitations of this study should be acknowledged. First, as a foundational engineering report focused on established standardized workflows and *in vitro* characterization, this study did not include *in vivo* grafting or long-term tissue integration assessments. While our transcriptomic data did not reveal significant activation of primary inflammatory pathways in MSCs, more specialized immunomodulation assays or profiling a broader panel of inflammatory cytokines will be essential to fully confirm the immunocompatibility of the allogenic scaffold before its progression to veterinary clinical trials. Second, we did not perform exact quantification of white blood cells or platelets in the starting platelet-rich plasma batches. Given that variations in cellular counts can influence growth factor profiles and gel kinetics, establishing standardized batch-release criteria will be a priority for future translational studies. Third, while our diffusion assay demonstrated size-selective transport, the use of commercial inks provided primarily qualitative data, future research should employ defined molecular weight markers, such as FITC-dextrans, to quantitatively map the effective pore size and permeability of the matrix. Fourth, we recognize the value of comparing PFMG with other commercially available porcine-derived scaffolds (e.g., collagen gels). Such side-by-side comparisons will help refine the specific therapeutic scenarios where fibrin-based systems offer superior clinical outcomes. Finally, the relatively lower apparent differentiation efficiency in the mixed configuration remains a technical constraint. Future efforts will focus on optimizing induction protocols for fully embedded cells or integrating dynamic mechanical stimuli to enhance lineage-specific signaling in 3D. Addressing these aspects will strengthen the case for PFMG as a robust, clinical-grade biomaterial for regenerative medicine.

## 5 Conclusion

In this study, we developed porcine fibrin matrix gel (PFMG) as a novel 3D cell culture scaffold derived from whole porcine blood. Compared with traditional 2D culture media, PFMG significantly increased the proliferation of porcine mesenchymal stem cells and embryonic fibroblasts, providing a more supportive environment for cell growth. PFMG also supported the full tri-lineage (adipogenic, osteogenic, chondrogenic) differentiation of MSCs, with adipogenic differentiation being more efficient in PFMG when supplemented with PPL than with FBS. These findings suggest that PFMG is a promising scaffold material for tissue engineering and regenerative medicine, offering a cost-effective and reproducible alternative to conventional FBS-based culture systems.

## Data Availability

The original contributions presented in the study are included in the article/supplementary material, further inquiries can be directed to the corresponding author.
